# Dichlorido(η^4^-cyclo­octa-1,5-diene)bis­(propane­nitrile-κ*N*)ruthenium(II)

**DOI:** 10.1107/S1600536811035379

**Published:** 2011-09-14

**Authors:** Haleden Chiririwa, Reinout Meijboom

**Affiliations:** a Research Centre for Synthesis and Catalysis, Department of Chemistry, University of Johannesburg, PO Box 524, Auckland Park, 2006 Johannesburg, South Africa

## Abstract

In the title complex, [RuCl_2_(C_8_H_12_)(C_3_H_5_N)_2_], the metal ion is coordinated to both double bonds of the cyclo­octa-1,5-diene ligand, two chloride ions (in *cis* positions) and two N-atom donors from two propane­nitrile mol­ecules that complete the coordination sphere for the neutral complex. The coordination around the Ru^II^ atom can thus be considered as octa­hedral with slight trigonal distortion.

## Related literature

For the structure of the acetonitrile derivative, see: Ashworth *et al.* (1987 ▶); Chiririwa *et al.* (2011 ▶). For the synthesis of starting materials, see: Ashworth *et al.* (1987 ▶).
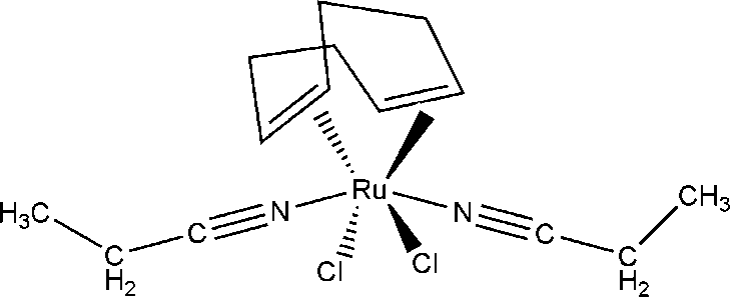


## Experimental

### 

#### Crystal data

[RuCl_2_(C_8_H_12_)(C_3_H_5_N)_2_]
*M* *_r_* = 390.31
Triclinic, 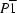

*a* = 7.593 (5) Å
*b* = 8.800 (5) Å
*c* = 12.658 (5) Å
α = 108.156 (5)°
β = 96.281 (5)°
γ = 90.536 (5)°
*V* = 798.0 (8) Å^3^
*Z* = 2
Mo *K*α radiation
μ = 1.31 mm^−1^
*T* = 100 K
0.29 × 0.28 × 0.21 mm

#### Data collection

Bruker APEXII CCD diffractometer
15438 measured reflections
3949 independent reflections
3911 reflections with *I* > 2σ(*I*)
*R*_int_ = 0.029

#### Refinement

*R*[*F* ^2^ > 2σ(*F* ^2^)] = 0.017
*wR*(*F* ^2^) = 0.043
*S* = 1.07
3949 reflections
174 parameters
H-atom parameters constrained
Δρ_max_ = 0.55 e Å^−3^
Δρ_min_ = −0.62 e Å^−3^

### 

Data collection: *APEX2* (Bruker, 2007 ▶); cell refinement: *SAINT-Plus* (Bruker, 2007 ▶); data reduction: *SAINT-Plus* and *XPREP* (Bruker, 2007 ▶); program(s) used to solve structure: *SHELXS97* (Sheldrick, 2008 ▶); program(s) used to refine structure: *SHELXL97* (Sheldrick, 2008 ▶); molecular graphics: *DIAMOND* (Brandenburg & Putz, 2005 ▶) and *ORTEP-3* (Farrugia, 1997 ▶); software used to prepare material for publication: *WinGX* (Farrugia, 1999 ▶).

## Supplementary Material

Crystal structure: contains datablock(s) global, I. DOI: 10.1107/S1600536811035379/im2311sup1.cif

Structure factors: contains datablock(s) I. DOI: 10.1107/S1600536811035379/im2311Isup2.hkl

Additional supplementary materials:  crystallographic information; 3D view; checkCIF report

## Figures and Tables

**Fig. 1.**
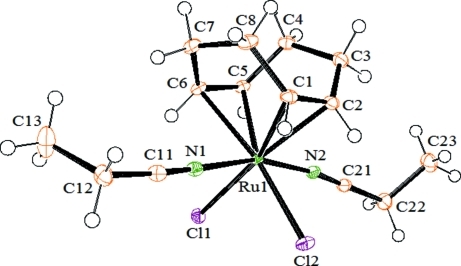
Molecular structure of the title compound, showing 50% probability displacement ellipsoids.

## supplementary crystallographic information

### Comment

The present ruthenium complex, Fig.1, has been synthesized in a similar way as done earlier for the acetonitrile derivative (Chiririwa *et al.* 2011). Organonitrile solvate complexes are widely useful for synthesis of organometallic compounds because of facile substitution at the solvate coordination sites. Similarly, 1,5-cyclooctadiene complexes have found considerable use in organometallic chemistry as well.

The two propanenitrile ligands are *trans* to each other, although the N(2)—Ru(1)—N(1) angle is widened to 164.95 (5)° due to repulsion by the alkene bonds of the COD ligand. The corresponding angle is 163.15 (6)° in the acetonitrile derivative. One of the propanenitrile ligands is slightly bent as we observed earlier in the acetonitrile derivative. The N(2)—C(21)—C(22) bond angle is 176.8 (2)°. The C(21)—C(22)—C(23) and C(11)—C(12)—C(13) bond angles are slightly bigger than the ideal tetrahedral angle and are almost similar with values of 111.3 (1)° and 111.8 (1)° respectively.

### Experimental

A suspension of [{RuCl_2_(COD)}*_x_*] (0.5 g) in propanenitrile (30 ml) was refluxed for 8 h. The orange solution was filtered hot and concentrated on a steam bath to *ca.* half volume. Cooling to 0 °C overnight afforded orange crystals suitable for X-ray diffraction studies in 50% yield.

### Refinement

The methylene and methyl H atoms were placed in geometrically idealized positions (C—H = 0.95–0.98) and constrained to ride on their parent atoms with *U*_iso_(H) = 1.2*U*_eq_(C) for methylene H atoms, and *U*_iso_(H) = 1.5*U*_eq_(C) for methyl H atoms respectively.

### Crystal data

**Table:**

[RuCl_2_(C_8_H_12_)(C_3_H_5_N)_2_]	*Z* = 2
*M**_r_* = 390.31	*F*(000) = 396
Triclinic, *P*1	*D*_x_ = 1.624 Mg m^−^^3^
Hall symbol: -P 1	Mo *K*α radiation, λ = 0.71069 Å
*a* = 7.593 (5) Å	Cell parameters from 9962 reflections
*b* = 8.800 (5) Å	θ = 3.0–28.3°
*c* = 12.658 (5) Å	µ = 1.31 mm^−^^1^
α = 108.156 (5)°	*T* = 100 K
β = 96.281 (5)°	Block, orange
γ = 90.536 (5)°	0.29 × 0.28 × 0.21 mm
*V* = 798.0 (8) Å^3^	

### Data collection

**Table:**

Bruker APEXII CCD diffractometer	3911 reflections with *I* > 2σ(*I*)
Radiation source: fine-focus sealed tube	*R*_int_ = 0.029
graphite	θ_max_ = 28.3°, θ_min_ = 1.7°
φ and ω scans	*h* = −10→10
15438 measured reflections	*k* = −11→10
3949 independent reflections	*l* = −15→16

### Refinement

**Table:**

Refinement on *F*^2^	Primary atom site location: structure-invariant direct methods
Least-squares matrix: full	Secondary atom site location: difference Fourier map
*R*[*F*^2^ > 2σ(*F*^2^)] = 0.017	Hydrogen site location: inferred from neighbouring sites
*wR*(*F*^2^) = 0.043	H-atom parameters constrained
*S* = 1.07	*w* = 1/[σ^2^(*F*_o_^2^) + (0.0132*P*)^2^ + 0.6531*P*] where *P* = (*F*_o_^2^ + 2*F*_c_^2^)/3
3949 reflections	(Δ/σ)_max_ = 0.002
174 parameters	Δρ_max_ = 0.55 e Å^−^^3^
0 restraints	Δρ_min_ = −0.62 e Å^−^^3^

### Special details

**Table:**

Geometry. All e.s.d.'s (except the e.s.d. in the dihedral angle between two l.s. planes) are estimated using the full covariance matrix. The cell e.s.d.'s are taken into account individually in the estimation of e.s.d.'s in distances, angles and torsion angles; correlations between e.s.d.'s in cell parameters are only used when they are defined by crystal symmetry. An approximate (isotropic) treatment of cell e.s.d.'s is used for estimating e.s.d.'s involving l.s. planes.
Refinement. Refinement of *F*^2^ against ALL reflections. The weighted *R*-factor *wR* and goodness of fit *S* are based on *F*^2^, conventional *R*-factors *R* are based on *F*, with *F* set to zero for negative *F*^2^. The threshold expression of *F*^2^ > σ(*F*^2^) is used only for calculating *R*-factors(gt) *etc*. and is not relevant to the choice of reflections for refinement. *R*-factors based on *F*^2^ are statistically about twice as large as those based on *F*, and *R*- factors based on ALL data will be even larger.

### Fractional atomic coordinates and isotropic or equivalent isotropic displacement parameters (Å^2^)

**Table:**

	*x*	*y*	*z*	*U*_iso_*/*U*_eq_	
C1	−0.11889 (18)	0.24062 (16)	0.86381 (11)	0.0132 (2)	
H1	−0.0776	0.3394	0.9141	0.016*	
C2	−0.21829 (18)	0.23779 (16)	0.76462 (12)	0.0134 (2)	
H2	−0.2427	0.3352	0.7532	0.016*	
C3	−0.28956 (19)	0.08642 (17)	0.67378 (12)	0.0159 (3)	
H3A	−0.3375	0.114	0.6083	0.019*	
H3B	−0.3862	0.04	0.6995	0.019*	
C4	−0.14983 (19)	−0.04040 (16)	0.63958 (12)	0.0147 (3)	
H4A	−0.1537	−0.1125	0.6838	0.018*	
H4B	−0.1805	−0.103	0.5616	0.018*	
C5	0.03841 (18)	0.03064 (15)	0.65508 (11)	0.0121 (2)	
H5	0.0783	0.0616	0.5976	0.015*	
C6	0.15194 (18)	0.05054 (15)	0.75234 (11)	0.0125 (2)	
H6	0.2672	0.0906	0.7565	0.015*	
C7	0.09657 (19)	0.00994 (16)	0.85206 (12)	0.0151 (3)	
H7A	0.1929	0.0421	0.9126	0.018*	
H7B	0.0776	−0.1052	0.8317	0.018*	
C8	−0.0737 (2)	0.09040 (17)	0.89491 (12)	0.0161 (3)	
H8A	−0.1728	0.0129	0.8656	0.019*	
H8B	−0.0608	0.1179	0.9758	0.019*	
C11	0.30431 (19)	0.35812 (17)	0.98608 (12)	0.0154 (3)	
C12	0.4147 (2)	0.37569 (18)	1.09193 (12)	0.0177 (3)	
H12A	0.339	0.3788	1.1494	0.021*	
H12B	0.4839	0.4762	1.1147	0.021*	
C13	0.5395 (2)	0.2387 (2)	1.08180 (15)	0.0295 (4)	
H13A	0.4716	0.1407	1.0687	0.044*	
H13B	0.6184	0.2599	1.1499	0.044*	
H13C	0.6072	0.2291	1.0205	0.044*	
C21	−0.12818 (18)	0.32906 (16)	0.52863 (11)	0.0132 (2)	
C22	−0.2237 (2)	0.37027 (19)	0.43512 (12)	0.0181 (3)	
H22A	−0.1637	0.3277	0.3689	0.022*	
H22B	−0.2228	0.4858	0.453	0.022*	
C23	−0.4155 (2)	0.3029 (2)	0.41046 (14)	0.0225 (3)	
H23A	−0.4167	0.1881	0.384	0.034*	
H23B	−0.4781	0.341	0.3543	0.034*	
H23C	−0.472	0.3374	0.4777	0.034*	
N1	0.22016 (16)	0.33593 (14)	0.90225 (10)	0.0129 (2)	
N2	−0.05984 (15)	0.30029 (13)	0.60433 (10)	0.0119 (2)	
Cl1	0.33515 (4)	0.32622 (4)	0.67591 (3)	0.01333 (7)	
Cl2	0.01618 (5)	0.57453 (4)	0.81456 (3)	0.01527 (7)	
Ru1	0.065082 (13)	0.290208 (11)	0.752556 (8)	0.00846 (4)	

### Atomic displacement parameters (Å^2^)

**Table:**

	*U*^11^	*U*^22^	*U*^33^	*U*^12^	*U*^13^	*U*^23^
C1	0.0144 (6)	0.0121 (6)	0.0140 (6)	−0.0003 (5)	0.0058 (5)	0.0043 (5)
C2	0.0118 (6)	0.0124 (6)	0.0170 (6)	0.0000 (5)	0.0043 (5)	0.0051 (5)
C3	0.0133 (6)	0.0147 (6)	0.0187 (6)	−0.0016 (5)	0.0014 (5)	0.0041 (5)
C4	0.0167 (6)	0.0120 (6)	0.0144 (6)	−0.0016 (5)	0.0013 (5)	0.0026 (5)
C5	0.0153 (6)	0.0089 (6)	0.0128 (6)	0.0013 (5)	0.0028 (5)	0.0036 (5)
C6	0.0158 (6)	0.0088 (6)	0.0139 (6)	0.0016 (5)	0.0025 (5)	0.0047 (5)
C7	0.0200 (7)	0.0128 (6)	0.0144 (6)	0.0010 (5)	0.0016 (5)	0.0073 (5)
C8	0.0226 (7)	0.0146 (6)	0.0142 (6)	0.0005 (5)	0.0056 (5)	0.0077 (5)
C11	0.0175 (7)	0.0137 (6)	0.0153 (6)	−0.0010 (5)	0.0027 (5)	0.0047 (5)
C12	0.0179 (7)	0.0207 (7)	0.0129 (6)	−0.0012 (5)	−0.0017 (5)	0.0041 (5)
C13	0.0230 (8)	0.0353 (9)	0.0248 (8)	0.0108 (7)	−0.0039 (6)	0.0039 (7)
C21	0.0141 (6)	0.0112 (6)	0.0147 (6)	−0.0006 (5)	0.0019 (5)	0.0044 (5)
C22	0.0171 (7)	0.0238 (7)	0.0169 (6)	0.0001 (5)	−0.0019 (5)	0.0129 (6)
C23	0.0172 (7)	0.0272 (8)	0.0218 (7)	−0.0018 (6)	−0.0044 (6)	0.0084 (6)
N1	0.0158 (5)	0.0117 (5)	0.0119 (5)	−0.0009 (4)	0.0018 (4)	0.0045 (4)
N2	0.0128 (5)	0.0101 (5)	0.0130 (5)	0.0004 (4)	0.0019 (4)	0.0039 (4)
Cl1	0.01152 (14)	0.01657 (15)	0.01382 (14)	0.00017 (11)	0.00207 (11)	0.00737 (12)
Cl2	0.02060 (16)	0.00875 (14)	0.01670 (15)	0.00064 (11)	0.00411 (12)	0.00369 (11)
Ru1	0.01012 (6)	0.00786 (6)	0.00801 (6)	0.00031 (4)	0.00092 (4)	0.00342 (4)

### Geometric parameters (Å, °)

**Table:**

C1—C2	1.386 (2)	C8—H8A	0.97
C1—C8	1.524 (2)	C8—H8B	0.97
C1—Ru1	2.2197 (15)	C11—N1	1.1368 (19)
C1—H1	0.93	C11—C12	1.465 (2)
C2—C3	1.512 (2)	C12—C13	1.523 (2)
C2—Ru1	2.2278 (19)	C12—H12A	0.97
C2—H2	0.93	C12—H12B	0.97
C3—C4	1.543 (2)	C13—H13A	0.96
C3—H3A	0.97	C13—H13B	0.96
C3—H3B	0.97	C13—H13C	0.96
C4—C5	1.522 (2)	C21—N2	1.1387 (19)
C4—H4A	0.97	C21—C22	1.4639 (19)
C4—H4B	0.97	C22—C23	1.529 (2)
C5—C6	1.386 (2)	C22—H22A	0.97
C5—Ru1	2.2274 (17)	C22—H22B	0.97
C5—H5	0.93	C23—H23A	0.96
C6—C7	1.5140 (19)	C23—H23B	0.96
C6—Ru1	2.2150 (17)	C23—H23C	0.96
C6—H6	0.93	N1—Ru1	2.0413 (14)
C7—C8	1.549 (2)	N2—Ru1	2.0356 (14)
C7—H7A	0.97	Cl1—Ru1	2.4211 (13)
C7—H7B	0.97	Cl2—Ru1	2.4231 (14)
			
C2—C1—C8	123.45 (12)	C11—C12—H12B	109.3
C2—C1—Ru1	72.16 (9)	C13—C12—H12B	109.3
C8—C1—Ru1	112.17 (9)	H12A—C12—H12B	107.9
C2—C1—H1	118.3	C12—C13—H13A	109.5
C8—C1—H1	118.3	C12—C13—H13B	109.5
Ru1—C1—H1	85.7	H13A—C13—H13B	109.5
C1—C2—C3	124.17 (13)	C12—C13—H13C	109.5
C1—C2—Ru1	71.53 (8)	H13A—C13—H13C	109.5
C3—C2—Ru1	111.07 (9)	H13B—C13—H13C	109.5
C1—C2—H2	117.9	N2—C21—C22	176.82 (15)
C3—C2—H2	117.9	C21—C22—C23	111.33 (13)
Ru1—C2—H2	87.4	C21—C22—H22A	109.4
C2—C3—C4	113.93 (12)	C23—C22—H22A	109.4
C2—C3—H3A	108.8	C21—C22—H22B	109.4
C4—C3—H3A	108.8	C23—C22—H22B	109.4
C2—C3—H3B	108.8	H22A—C22—H22B	108
C4—C3—H3B	108.8	C22—C23—H23A	109.5
H3A—C3—H3B	107.7	C22—C23—H23B	109.5
C5—C4—C3	113.59 (12)	H23A—C23—H23B	109.5
C5—C4—H4A	108.8	C22—C23—H23C	109.5
C3—C4—H4A	108.8	H23A—C23—H23C	109.5
C5—C4—H4B	108.8	H23B—C23—H23C	109.5
C3—C4—H4B	108.8	C11—N1—Ru1	178.21 (12)
H4A—C4—H4B	107.7	C21—N2—Ru1	170.17 (11)
C6—C5—C4	121.89 (12)	N2—Ru1—N1	164.95 (5)
C6—C5—Ru1	71.34 (8)	N2—Ru1—C6	115.27 (5)
C4—C5—Ru1	113.47 (9)	N1—Ru1—C6	76.54 (5)
C6—C5—H5	119.1	N2—Ru1—C1	113.39 (6)
C4—C5—H5	119.1	N1—Ru1—C1	76.63 (6)
Ru1—C5—H5	85.4	C6—Ru1—C1	80.71 (6)
C5—C6—C7	122.86 (13)	N2—Ru1—C5	79.67 (5)
C5—C6—Ru1	72.32 (8)	N1—Ru1—C5	112.85 (5)
C7—C6—Ru1	111.05 (9)	C6—Ru1—C5	36.35 (5)
C5—C6—H6	118.6	C1—Ru1—C5	87.81 (5)
C7—C6—H6	118.6	N2—Ru1—C2	77.11 (5)
Ru1—C6—H6	86.7	N1—Ru1—C2	112.46 (6)
C6—C7—C8	114.14 (11)	C6—Ru1—C2	94.63 (6)
C6—C7—H7A	108.7	C1—Ru1—C2	36.30 (5)
C8—C7—H7A	108.7	C5—Ru1—C2	79.57 (5)
C6—C7—H7B	108.7	N2—Ru1—Cl1	84.91 (5)
C8—C7—H7B	108.7	N1—Ru1—Cl1	86.21 (5)
H7A—C7—H7B	107.6	C6—Ru1—Cl1	88.31 (4)
C1—C8—C7	115.40 (11)	C1—Ru1—Cl1	161.35 (4)
C1—C8—H8A	108.4	C5—Ru1—Cl1	92.24 (4)
C7—C8—H8A	108.4	C2—Ru1—Cl1	161.29 (4)
C1—C8—H8B	108.4	N2—Ru1—Cl2	83.36 (4)
C7—C8—H8B	108.4	N1—Ru1—Cl2	85.00 (4)
H8A—C8—H8B	107.5	C6—Ru1—Cl2	161.36 (4)
N1—C11—C12	176.32 (15)	C1—Ru1—Cl2	92.67 (4)
C11—C12—C13	111.76 (13)	C5—Ru1—Cl2	161.68 (4)
C11—C12—H12A	109.3	C2—Ru1—Cl2	90.02 (4)
C13—C12—H12A	109.3	Cl1—Ru1—Cl2	93.06 (2)
			
C8—C1—C2—C3	−1.8 (2)	C8—C1—Ru1—C6	8.48 (10)
Ru1—C1—C2—C3	103.38 (13)	C2—C1—Ru1—C5	−75.28 (9)
C8—C1—C2—Ru1	−105.22 (13)	C8—C1—Ru1—C5	44.33 (10)
C1—C2—C3—C4	−49.30 (18)	C8—C1—Ru1—C2	119.61 (13)
Ru1—C2—C3—C4	32.14 (15)	C2—C1—Ru1—Cl1	−165.82 (9)
C2—C3—C4—C5	−28.89 (17)	C8—C1—Ru1—Cl1	−46.21 (17)
C3—C4—C5—C6	93.14 (16)	C2—C1—Ru1—Cl2	86.39 (8)
C3—C4—C5—Ru1	11.23 (15)	C8—C1—Ru1—Cl2	−154.00 (9)
C4—C5—C6—C7	−2.5 (2)	C6—C5—Ru1—N2	168.49 (9)
Ru1—C5—C6—C7	104.06 (12)	C4—C5—Ru1—N2	−74.04 (10)
C4—C5—C6—Ru1	−106.56 (12)	C6—C5—Ru1—N1	−2.75 (9)
C5—C6—C7—C8	−53.39 (18)	C4—C5—Ru1—N1	114.72 (10)
Ru1—C6—C7—C8	28.63 (14)	C4—C5—Ru1—C6	117.46 (13)
C2—C1—C8—C7	87.27 (17)	C6—C5—Ru1—C1	−77.26 (9)
Ru1—C1—C8—C7	4.58 (15)	C4—C5—Ru1—C1	40.21 (10)
C6—C7—C8—C1	−22.28 (17)	C6—C5—Ru1—C2	−112.87 (9)
C5—C6—Ru1—N2	−12.53 (9)	C4—C5—Ru1—C2	4.60 (10)
C7—C6—Ru1—N2	−131.71 (10)	C6—C5—Ru1—Cl1	84.08 (9)
C5—C6—Ru1—N1	177.40 (9)	C4—C5—Ru1—Cl1	−158.46 (9)
C7—C6—Ru1—N1	58.22 (10)	C6—C5—Ru1—Cl2	−169.16 (9)
C5—C6—Ru1—C1	99.03 (9)	C4—C5—Ru1—Cl2	−51.69 (17)
C7—C6—Ru1—C1	−20.15 (10)	C1—C2—Ru1—N2	−177.67 (9)
C7—C6—Ru1—C5	−119.18 (14)	C3—C2—Ru1—N2	61.93 (10)
C5—C6—Ru1—C2	65.39 (9)	C1—C2—Ru1—N1	−9.89 (9)
C7—C6—Ru1—C2	−53.79 (10)	C3—C2—Ru1—N1	−130.28 (10)
C5—C6—Ru1—Cl1	−96.10 (8)	C1—C2—Ru1—C6	67.44 (8)
C7—C6—Ru1—Cl1	144.72 (9)	C3—C2—Ru1—C6	−52.95 (11)
C5—C6—Ru1—Cl2	169.34 (9)	C3—C2—Ru1—C1	−120.39 (14)
C7—C6—Ru1—Cl2	50.16 (18)	C1—C2—Ru1—C5	100.66 (9)
C2—C1—Ru1—N2	2.47 (9)	C3—C2—Ru1—C5	−19.73 (10)
C8—C1—Ru1—N2	122.09 (10)	C1—C2—Ru1—Cl1	165.87 (9)
C2—C1—Ru1—N1	170.61 (9)	C3—C2—Ru1—Cl1	45.47 (18)
C8—C1—Ru1—N1	−69.77 (10)	C1—C2—Ru1—Cl2	−94.49 (8)
C2—C1—Ru1—C6	−111.14 (9)	C3—C2—Ru1—Cl2	145.12 (10)
